# Mellitic Acid-Supported Synthesis of Anisotropic Nanoparticles Used as SERS Substrate

**DOI:** 10.1021/acsomega.4c04592

**Published:** 2024-07-27

**Authors:** Beata Wrzosek, Karolina Zajdel, Paulina Jeleń, Jolanta Bukowska

**Affiliations:** † University of Warsaw, Faculty of Chemistry, Pasteura 1, 02-093 Warsaw, Poland; ‡ NOMATEN Centre of Excellence, National Centre for Nuclear Research, 7 Andrzeja Sołtana Street, 05-400 Otwock, Poland; § Electron Microscopy Research Unit, Polish Academy of Sciences, Mossakowski Medical Research Institute, 5 Pawińskiego Street, 02-106 Warsaw, Poland

## Abstract

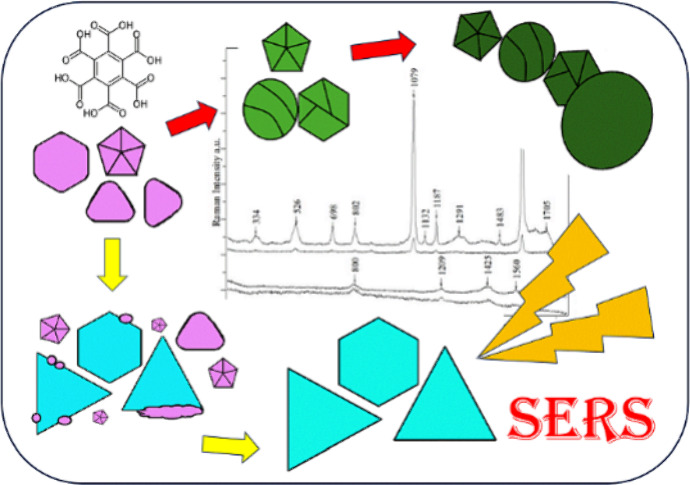

A method for the synthesis of a new SERS substrate—anisotropic silver nanoparticles using mellitic acid as a new capping agent is presented. The synthesis is free of toxic substances and does not require special temperature or lighting conditions. Moreover, it is fast, easy, and inexpensive. Depending on the concentration of silver ions and nanoparticle seeds, four different colloids were obtained, representing the evolution of nanoparticle growth along different paths from the first common stage. One of the synthesized colloids consists mainly of triangular nanoplates, while the other consists of polyhedral NPs. The analysis of the synthesis process together with the observation of TEM images and UV–vis extinction spectra enabled the proposal of the mechanism of interaction of mellitic acid molecules as the capping agent. The ability of mellitic acid molecules to form a hydrogen bond network, together with a ratio of silver ions to the mellitic acid concentration, turned out to be crucial for determining the shape of the NPs. All obtained colloids strongly enhance the Raman spectra of analyte molecules, thus proving their applicability as efficient new SERS substrates. For the one that enhanced the spectra the most, the detection limit was set at 10^–9^ M. Using it as a SERS substrate enables the identification of a trace amount of a designer drug, i.e., 4-chloromethcathinone (4-CMC, clephedrone). For the first time, SERS spectra of this substance, illegal in many countries, are presented.

## Introduction

Surface enhanced raman scattering (SERS) is a well-known analytical method that requires the use of nanometer-sized metallic clusters with plasmonic properties.^1−11^ The enhancement of the electromagnetic field (EM) around metallic nanoparticles (NPs) is a direct consequence of resonance associated with coherent oscillations of the surface conduction electrons. Usually, NPs used as a SERS substrate are aggregated, and the strongest electromagnetic field responsible for SERS enhancement is found at the junctions of the nanoparticles.^12−19^ For anisotropic NPs, the distribution of the electromagnetic field enhancement is strongly heterogeneous, even around a single nanoparticle with the maximum value areas on its edges and tops.^10,19−21^ The places of superior EM enhancement, in the junction or in the edges, are called hot spots. The enhancement of the EM field in the junction of aggregated nanoparticles can be strong enough to trap a single molecule^22^ and allow its SERS spectrum to be recorded^23,24^ accompanied by the blinking effect,^25,26^ which in the case of anisotropic NPs can be additionally supported by the hot spots on the top/edges.^27^

The anisotropy of NPs results not only in a higher diversity of the hot spots but also, consequently, in a greater number of surface plasmon resonance (SPR) frequencies. The number of SPR bands and their positions in the UV–vis extinction spectrum depend strongly on the shape and size of NPs, and their diversity enables tuning to the needs of the detection of a given compound/system.^10,18,28−38^ The application field of anisotropic NPs extends over many areas of research, beginning from physicochemical processes such as electro- and photocatalysis to optical analytical methods such as surface enhanced infrared absorption (SEIRA) and surface enhanced raman scattering (SERS), and metal enhanced fluorescence (MEF), and utilization in biomedical detection, ending with the photothermal therapy.^39−48^

One of the most commonly used methods for obtaining anisotropic nanoparticles is seed-mediated growth synthesis.^45,47,49−53^ In the first part of the synthesis, small, spherical nanoparticles, called “seeds”, are obtained. In the next step, an specified amount of seeds is added to a solution containing silver ions, a capping agent, and a reducing agent. To prevent the reduction of silver ions before the addition of Ag seeds, a rather weak reducing agent is used (e.g., ascorbic acid). Surfactants are typically employed as capping agents.

There are many theories trying to explain the formation mechanism of anisotropic NPs.^47,49−51,54−57^ Some explain it by the interaction of surfactants with metal salts, which results in the growth of metal crystals on surfaces or inside micelles/reverse micelles of aggregated amphiphilic molecules, using them as a physical template. Another hypothesis assumes that surfactant molecules are selectively adsorbed (through van der Waals interactions) on the definite crystallographic facets of the metal seed, thus preventing their further growth. One more theory postulates that there are some crystallographic facets of the metal seed on which metal ions adsorb preferably; thus, they grow on them or/and prevent the growth of other metal ions coexisting in the solution.

Until now, the methods of synthesizing anisotropic Au NPs have been successfully developed and optimized, definitely better than the synthesis of Ag NPs.^45,46,52,58^ Nevertheless, an increasing number of applications of SERS require further development of silver substrates, as silver provides stronger enhancement of Raman spectra.^9^ Additionally, anisotropic Ag NPs with well-defined shapes (wires, cubes, bipyramids, and rods) have been obtained best from organic solutions, for example, in so-called polyol synthesis.^59−63^ However, the applicability of Ag NPs, for example, in SERS requires their preparation in aqueous solution. Until now, well-shaped Ag NPs (triangles, wires, and rods) have been successfully obtained in aqueous solutions by reducing silver ions with ascorbic acid on silver seeds in the alkaline solution in the presence of highly concentrated cetyltrimethylammonium bromide (CTAB).^64−66^ The success of this synthesis is, unfortunately, overshadowed by the high toxicity of CTAB.^58,67,68^ Murphy’s group presented the seedless, surfactantless, and aqueous synthesis of one-dimensional long Ag nanowires, where only sodium citrate and sodium hydroxide with silver salt were mixed at 100 °C. However, this substrate was applied more as a nanoscale conductor than a SERS support.^69,70^ Lu et al.^71^ described an aqueous synthesis where ammonium silver salt was reduced by ascorbic acid in the presence of citrate ions and PVP, but resultant NPs, despite their anisotropic structure, had rather rotund irregular tips (i.e., bulbous shaped). Finally, appropriately mixing AgNO_3_, trisodium citrate, NaBH_4_, H_2_O_2_, and PVP allowed Métraux and Mirkin to obtain excellent results: nanoprisms with controlled thickness.^72^ A disadvantage of this synthesis is the strong affinity of the polar pyrrolidone group of PVP for the Au or Ag surfaces, which hinders the replacement the PVP layer and functionalization of the NP surface by another compounds.^73,74^ The “green” recipe for obtaining well-shaped (spiky stars) NPs with AgNO_3_ reduction by ascorbic acid, without surfactant covers, has finally been developed.^73,75−77^ Nevertheless, Ag^+^ was not the only metal ion in this synthesis; AuCl_4_^–^ ions were also coreduced. Synthesis of Ag nanorods or sea-urchin-like NPs using Au seeds is, in fact, currently applied with great efficiency.^78−80^ A lot of studies done for the optimization of the synthesis of anisotropic Ag NPs had great results; nevertheless, there is still a hunger for aqueous, “green” synthesis not using surfactants strongly adsorbed on Ag or additional conditions like irradiation. Finally, Kochkar et al. described the synthesis that meets almost all conditions, in which ascorbic acid as a reducing agent and cyclodextrin (CD) as a capping agent were used.^81^ However, the authors did not test the obtained colloids as SERS substrates.

**Figure 1:**
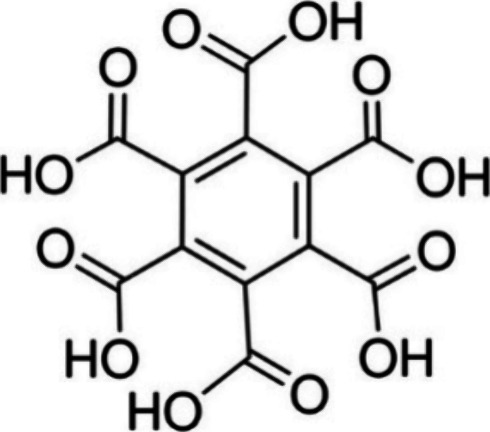
Chemical formula of mellitic acid (MA).

The described studies prompted us to apply another compound with similar features: highly symmetric molecules with a hydrophobic core surrounded by hydrophilic groups. It seemed that mellitic acid (MA) (Figure 1) could be a good candidate. MA is of high interest among scientists, as it has been shown that the oxidation of organic matter from Mars can lead to the formation of this acid.^82,83^ Until now, it has not been applied in the synthesis of NPs, and the idea that we present here to use it as a capping agent is new.

The presented results allowed us to determine the size and shape of NPs with the help of TEM images and UV–vis spectra. Moreover, by combining these results with an insightful analysis of the literature reports, we were able to understand the formation process of anisotropic NPs. Finally, the SERS experiments proved the excellent properties of our NPs as a SERS substrate.

## Experiment

### Materials

Trisodium citrate (CitNa_3_), silver nitrate, sodium borohydride, ascorbic acid, mellitic acid (MA), hydroxylamine hydrochloride, and 4-mercaptobenzoic acid (4-MBA) were purchased from Sigma-Aldrich. 4-Chloromethcathinone (4-CMC, clephedrone) was loaned from the Center for Forensic Sciences of the University of Warsaw.

### Methods

The morphology of the AgNPs was characterized using a JEM-1011 transmission electron microscope (JEOL, Tokyo, Japan) operating at 80 kV. The samples were prepared by spotting 1 μL of nanoparticle solution onto a Formvar-coated copper grid (Agar Scientific, London, UK) and air-drying the sample at room temperature by evaporating the solvent.

SERS spectra were recorded with a LabRAM HR800 (Horiba Jobin Yvon) Raman spectrometer equipped with a Peltier-cooled CCD detector (1024 × 256 pixels) coupled with an Olympus BX61 confocal microscope with a 50× long-distance objective. The measurements were carried out utilizing a diode-pumped frequency-doubled Nd:YAG laser of 532 nm wavelength and a He–Ne laser of 633 nm wavelength.

### Nanoparticle Preparation

#### Synthesis of Ag Seeds

In the first step, silver nitrate and trisodium citrate solutions were added to 5 mL of ice-cooled distilled water in the following proportion: AgNO_3_, *V* = 0.2 mL, *C* = 0.025 M; CitNa_3_, *V*= 0.16 mL, *C* = 0.1 M; and NaBH_4_, *V* = 0.1 mL, *C* = 0.025 M. In the next step, ice-cooled sodium borohydride solution was added to the vigorously stirred reaction mixture: 100 μL of NaBH_4_ in 10 portions of 10 μL.

#### Seed-Mediated Growth Synthesis of Ag NPs

500 μL of AgNO_3_ aqueous solution of a different concentration (0.25, 2.5, or 25 mM) and 100 μL of 0.1 M ascorbic acid solution were added to 5 mL of 5 mM MA solution. In the next step, to the reaction mixture kept under vigorous stirring, 200 μL (in MA4 500 μL) of 10-fold diluted colloid seeds prepared in the seed synthesis was added in 2 portions. The last step was the addition of 500 μL of a 0.1 M NaOH solution. Obtained colloids are termed MA-capped colloids hereinafter.

### SERS Test Measurement

SERS spectra of 1 mM aqueous solutions of 4-mercaptobenzoic acid (4-MBA) in the obtained MA-capped colloids and in the hydroxylamine-reduced AgNP reference colloids were recorded in the same conditions: 30 s acquisition time and 2 accumulation using two laser lines at −633 and 532 nm. Spectra were recorded immediately after the 4-MBA and colloids.

SERS spectra of 4-chloromethcathinone (4-CMC) were recorded in MA1 colloid samples mixed with an aqueous solution of 4-CMC at various concentrations in a ratio of 10:1, applied to glass, and allowed to dry. All spectra in the 4-chloromethcathinone test were collected for 300 s in 2 accumulations using the 633 nm line.

## Results

### Synthesis Process

**Figure 2:**
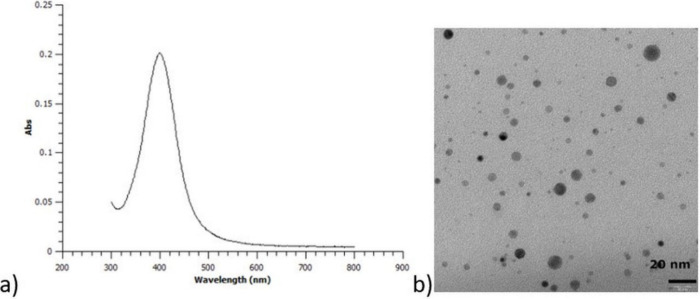
(a) UV–vis spectrum and (b) TEM image of the seeds.

The UV–vis spectrum of the seeds (Figure 2a) consists of a single asymmetric band with a maximum at 400 nm, which indicates the existence of only a dipole oscillating mode with absorption as a dominant effect. This points to a small size of nanoparticles (<10 nm) and a small size distribution, which are confirmed by TEM images (Figure 2 b).

After the seeds were added to the solution for seed-mediated growth synthesis, the reaction mixture turned yellow and changed over time to different colors depending on the Ag^+^ concentration.

At the lowest AgNO_3_ concentration, equal to 0.25 mM, the color evolution started from yellow, through orange, pink, and violet, and finally turned brown. If NaOH was added in the violet stage, no further color changes were observed. The colloid obtained in this way is referred to as MA1 violet hereinafter.

When the AgNO_3_ concentration was 10× higher (2.5 mM), a similar reaction was observed, but color changes did not stop at violet before turning brown. They continued to turn the solution blue, green, and then finally brown. The NaOH addition to the blue solution changed its color back to violet, and after a while it turned blue again, this time with a turquoise hue. This colloid is named MA2 turquoise.

When the AgNO_3_ concentration increased to 25 mM, the color of the solution changed from yellow to orange, pink, violet, and finally to darker violet before turning brown. When NaOH was added at the violet stage, the violet color rapidly changed into dark orange/warm brown and developed into turbid green. The same changes were observed when the amount of seeds increased 2.5×. The difference between those two colloids was only the hue of the final green color, which was darker in the second one. Those colloids are referred to as MA3 light green and MA4 dark green.

After repeating all of the above-described syntheses several times, four types of final Ag colloid products were confirmed and identified: MA1 violet, MA2 turquoise, MA3 light green, and MA4 dark turbid green.

### TEM and Histograms

TEM images of the colloids could only be recorded in their final form after the addition of NaOH. Not adding NaOH to the syntheses causes further reactions, and transformations (turning brown) ended with aggregation and the precipitation of aggregated nanoparticles. Dropping the sample onto the TEM mesh without adding NaOH to the synthesis caused the same processes to occur on the TEM mesh.

**Figure 3:**
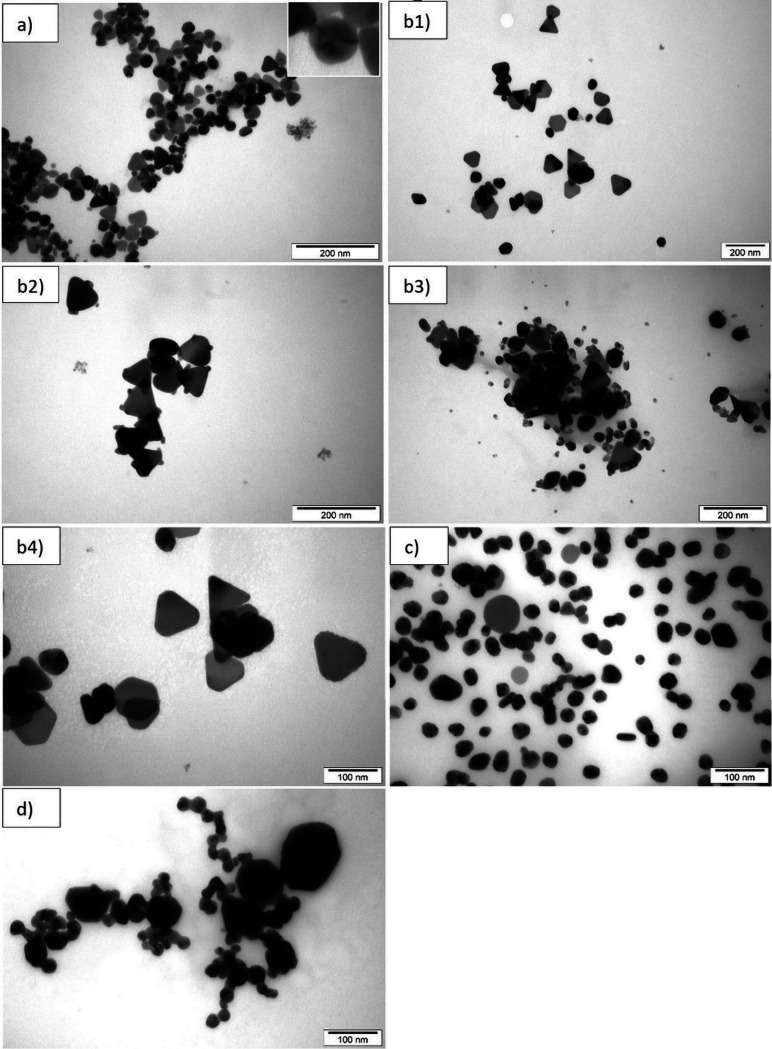
TEM images of colloids in a stable final stage after NaOH addition: (a) MA1 violet, (b1–b4) MA2 turquoise at different parts on the TEM grid, (c) MA3 light green, and (d) MA4 dark green.

**Figure 4:**
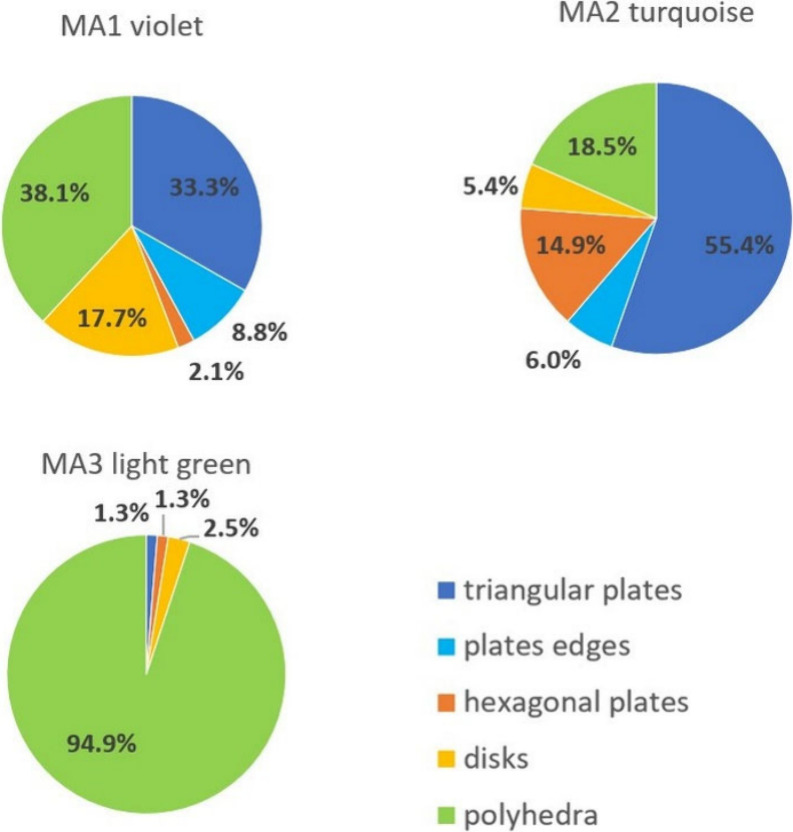
Shape distribution of nanoparticles in the obtained colloids.

**Figure 5:**
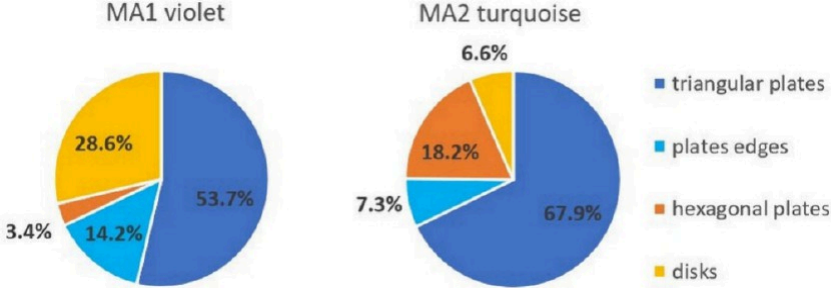
Shape distribution among plates in the MA1 and MA2 colloids.

The TEM images of the violet MA1 colloid (Figure 3) reveal two types of NPs. In this case, plates (disks, triangular and hexagonal plates, and plate edges) of similar dimensions of approximately 30–45 nm prevail (62%). Moreover, a large number of polyhedra with diameters of 20–30 nm (38%) are observed (histogram in Figure 4). The shape of 33.3% of all NPs and 53.7% of only plates (Figure 5) is recognized as triangular, 2.1% are hexagonal, and 17.7% are disks. The tops and edges of these polygonal plates are rounded but well-defined enough to be able to determine the shape of the respective plates. Some nanoplates lie more perpendicularly on the surface of the TEM mesh, which makes their edges visible from the front, and resemble nanorods in the TEM images. They are recognized as plate edges in 8.8% of all NPs. This arrangement of the nanoplates enables the determination of their thickness, which equals approximately 5 nm. The polyhedra, if recognized as regular, appear as 5 interconnected triangular faces (Figure 3a insert)). This feature indicates a decahedron,^84,85^ but an icosahedron is also possible.

All panels in Figure 3b1–b4 represent NPs from MA2 synthesis in a stable final stage after NaOH addition. They differ in the form of the addition to the larger NPs; in some of the SEM images, smaller NPs (Figure 3b2 and b3) can be seen, and in others another unshaped mass glued (Figure 3b3 and b4) to the larger NPs can be seen. Images without additional structures on the larger NPs were also recorded (Figure 3b1). All of the images in Figure 3b were recorded at transition after adding NaOH but from different places on the TEM grid and/or from separated samples of the product of the same synthesis repeated many times. Nanoparticles in the MA2 turquoise colloid are mostly plates, but their contribution is much higher (81.5%) in comparison with the MA1 violet colloid (Figure 4). They are also larger (75–90 nm), and polygonal plates have much better defined vertices and edges (Figure 3b1–b4). The contribution of disks to the total number of NPs is small (5.4%). The triangle is recognized as the shape of 55.4% of all NPs (Figure 4), which means an even greater percentage advantage over other shapes among plates (67.9%) in comparison with the MA1 violet colloid (Figure 5). The contribution of hexagonal plates increased to 14.9% of all NPs, and this is at the expense of decreasing the percentage of rounded disk-like nanoplates. Rod-shaped NPs, which are recognized as plate edges, occur here in a lower amount (6%). This is not surprising due to the larger size of the plates and hence the reduction of the tendency to dry vertically on their edges on the TEM mesh. In the case of this colloid, the vertical arrangement reveals a nanoplate thickness of approximately 10 nm. In this colloid, the content of the polyhedral NPs is only 18.5%, and they belong to the decahedra as in the case of the MA1 colloid. Their average diameter is about 60 nm. Additionally, a completely new feature of the MA2 colloid can be observed in comparison with the violet MA1 colloid, described above as very small spherical nanoparticles (<10 nm) (Figure 3b2 and b3) or a continuous mass of undefined shape but lumpy structure sticking on NPs of this colloid (Figure 3b3 and b4).

The TEM images of this light green colloid (Figure 3 c) show almost exclusively polyhedra with diameters of 25 nm. Some of them can be recognized as decahedra or icosahedra. Large plates (40–75 nm) are also visible but at a low percentage (5.1%) (Figure 4). A slight aggregation of NPs is also noticeable, whereas a significant degree of aggregation is visible in the TEM images of the MA4 colloid (Figure 3 d).

### UV–vis

**Figure 6:**
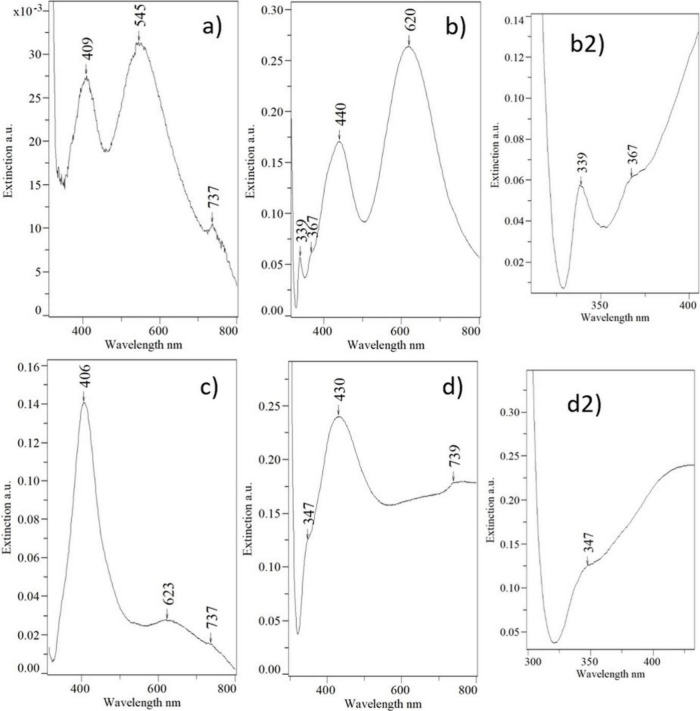
Extinction spectra of colloids: (a) MA1 violet, (b and b2) MA2 turquoise, (c) MA3 light green, and (d and d2) MA4 dark green.

The extinction spectrum of the MA1 violet colloid exhibits two intense and wide bands at 409 and 545 nm and a very weak band at 737 nm (Figure 6a). The first is usually assigned to the dipole mode of spherical NPs, but as the TEM images do not reveal such NPs in the MA1 colloid, this band probably results from polyhedral NPs with rounded tops and edges. The 545 nm band can be assigned to the dipole in-plane mode of triangle plates with truncated tops^10,28,29,33,36^ and hexagonal plates.^29,86,87^ Its broad shoulder from the shorter wavelength side hides a band with a maximum at around 500 nm, which corresponds to the dipole in-plane mode of decahedra.^88^ The 737 nm band usually appears in the extinction spectra of triangle nanoplate and is assigned to the in-plane dipole mode.^29,36^

In the extinction spectra of the MA2 colloid (Figure 6b), bands at 339, 368, 408, and 620 nm correspond to the quadrupole out-of-plane mode of triangle and hexagonal nanoplates, the dipole out-of-plane mode of hexagonal nanoplates, the dipole out-of-plane mode of triangle nanoplates, and the dipole in-plane mode of triangle and hexagonal nanoplates, respectively.^10,28,29,33,36,86,87,89^ It should be noted that the in-plane dipole band maximum (620 nm) is here shifted toward higher wavelengths with respect to the maximum of this band in the MA1 colloid spectrum (545 nm) (Figure 6a). This is consistent with the observation of the TEM images: the size of the nanoplates increased, and their tops and edges became sharper. The 620 nm band shoulder on the longer wavelength side originates from the triangle plates with even sharper tops, while the shoulder on the shorter wavelength side corresponds to less or more truncated triangle plates and hexagonal plates.^10,36,86,87^

What is interesting in this spectrum is the band at 440 nm. According to the literature data, a band around 460–480 nm can be found in the spectra of triangle and truncated triangle plates. It is assigned to the quadrupole in-plane mode.^10,36^ The band at 440 nm in the extinction spectrum of MA2 and the small spherical NPs and unshaped mass surrounding the nanoplates observed in the TEM images are new features of this colloid in comparison with MA1. The question is whether these features should be related to each other and this band should be assigned to a new resonance created in the junction of small spherical NPs and nanoplates or if this band should be just recognized as a blue shift from the typical wavelength of the quadrupole in-plane mode (460–480 nm).

The first hypothesis seems to be more correct, as a band with a maximum at 430 nm also appears as a strong feature in the extinction spectrum of the MA4 dark green colloid (Figure 6d), where almost all NPs are bonded to each other (Figure 3d). Besides this band, only weak 347 and 739 nm bands of anisotropic large nanoplates can be distinguished from the intense extinction background in the entire visible range. This intense background indicates the existence of many various types of modes. Some of them must be created in large-sized nanoparticles. The TEM images (Figure 3d) revealed that they are modes along a longer axis in the branch-shaped NPs.

In the extinction spectra of the light green MA3 colloid (Figure 6c), the 430–440 nm band is missing. This corresponds to the TEM images, which show the lack of such strong aggregation of NPs. It additionally proves that the 430–440 nm band in the spectra of MA2 and MA4 colloids originates from binding between NPs.

In the spectrum of the MA3 colloid, the 406 nm band dominates, which according to the TEM images (Figure 3c) should be assigned to polyhedral NPs with rounded tops and edges. The longer-wavelength shoulder probably conceals a band at 480 nm, assigned to decahedra with more developed edges and tops. Besides the 406 nm band, a broad but not intense band is observed with two slightly marked maxima at 623 and 737 nm, which should be assigned to the remainder of the large anisotropic nanoplates.

## Discussion

**Figure 7:**
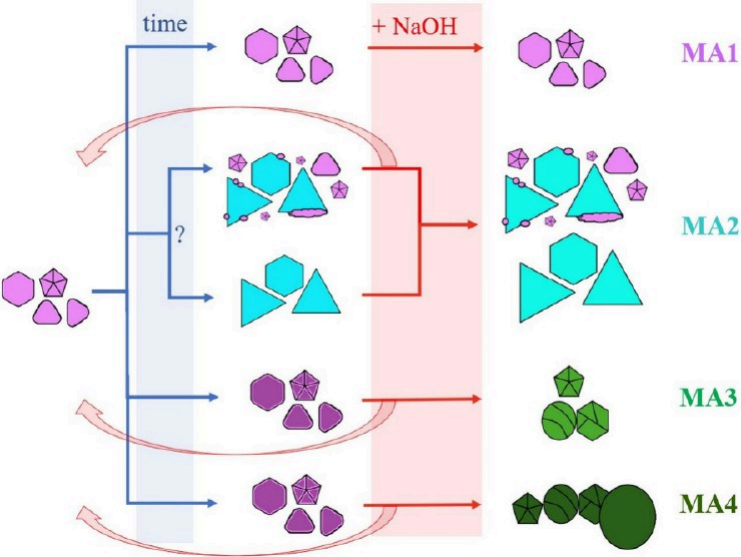
Scheme of the further evolution of MA1–MA4 syntheses after the initial common step.

The presented analysis of TEM images, histograms, and UV–vis spectra of obtained colloids shows how the change in the concentration of Ag^+^ determines the development of the synthesis path. The beginning of all syntheses seems to be the same: a violet colloid composed of polygonal nanoplates (mostly triangular) and polyhedra (decahedra and icosahedra) (Figure 7).

A 10× increase in the concentration of Ag^+^ ions (MA2 synthesis) leads to significant further changes: the appearance of a blue color, which is a result of the increasing size of the polygon nanoplates; sharpening of their tops; and an increase in the their population compared to polyhedra. Such developments of the synthesis indicate that it is dominated by the kinetic mechanism of nanoparticle growth.^90^ It is generally claimed that the differences in the growth rate of individual NP planes determine the final shape of nanoparticles and can be influenced by adsorption of capping agent molecules.^90−92^ In our syntheses, mellitic acid is the one that may be responsible for strongly inhibiting the growth of NPs in given planes and thus for the kinetic mechanism of nanoparticle growth and their anisotropic shape. At this point, the diversity in the crystallographic structure of the particular nanostructure facets must be discussed. Most of the final products of the MA2 synthesis were lamellar twinned particles–nanoplates, where the larger facets (top and bottom) are {111}, while lateral {100} facet for triangular nanoplates and mixed {111} and {100} facets for hexagonal nanoplates.^90^ Therefore, if mellitic acid is used as a capping agent, the results reveal a rather higher affinity of MA molecules to the {111} facets. This can be explained as follows: during the transition from the violet to the blue stage in MA2 synthesis, lateral {100} facets of nanoplates, which are free from the mellitic acid molecules, grow easier and faster. The dominance of the triangular nanoplates increases, and their tops become sharper. The percentage of the hexagonal nanoplates also increases but at the expense of the round disks. In contrast, decahedra and icosahedra are composed of only {111} facets; therefore, mellitic acid inhibits the growth and/or even the formation of NPs of these shapes in the MA1 and MA2 syntheses. Therefore, when the concentration ratio of mellitic acid to Ag^+^ was sufficiently low (MA3 and MA4 synthesis), MA was no longer able to inhibit the growth of {111} facets efficiently and promote the growth of nanoplates (lack of the blue stage, Figure 7).

It would also seem that a lower percentage of decahedra should be produced in the MA1 colloid as compared with the MA2 one, but the opposite is observed (Figure 4). It can be seen here that within these Ag^+^/MA concentration ratios, which are in the MA1 and MA2 syntheses, the absolute Ag^+^ concentration is decisive. Too low of a concentration of Ag ions in the MA1 synthesis prevents the dimensions of the NPs from increasing, i.e., prevents the development of the synthesis from the violet to the blue stage (Figure 7). In the MA2 synthesis containing a sufficient amount of Ag^+^, the increase in size can be observed for both decahedra and nanoplates (in diameter and thickness), but the contribution of decahedra decreases. This effect can be explained by the Ostwald ripening phenomena.^93^ A colloid as a system of nanoparticles dispersed in water tends to minimize interfacial energy. To decrease the interfacial area, some part of just expanded NP surfaces is dissolved back into the solution and rereduced on the biggest particles. In the process of NP growth (transition from the violet to the blue colloid), the polyhedral NPs could be those dissolving in favor of the growth of greater nanoplates. Adsorbed mellitic acid molecules can support this building material reorganization. MA molecules prefer to bind with hydrogen bonds on larger surfaces, so they relocate from the {111} faces of polyhedra to the MA network covering {111} facets on the top and bottom of nanoplates. During this process, MA molecules can detach Ag^+^ cations or Ag clusters from facets of polyhedra and attach to growing facets of nanoplates.

Another problem that needs discussion appeared in the last stage of the synthesis in which the pH was changed. Before the addition of NaOH, the colloids had a pH of about 2, and the addition of NaOH raised the pH only to about 3. The p*K*_a_ values of MA (at 25 °C) are 0.68, 2.21, 3.52, 5.09, 6.32, and 7.49 for p*K*_a_1–6, respectively, and those of ascorbic acid are 4.17 and 11.6 for p*K*_a_1 and p*K*_a_2. Therefore, the increase of the pH value from 2 to 3 does not significantly affect the protonation equilibrium of ascorbic acid. However, mellitic acid changes its form from a completely deprotonated acid at one carboxyl group to a completely deprotonated acid at two carboxyl groups. This decreased degree of protonation of mellitic acid does not change the final product MA2 to a very large extent. The addition of NaOH to the solution at the blue color stage in the MA2 synthesis ultimately enabled a colloid of a similar color to be obtained. Nevertheless, it could be seen that this was achieved by taking a step back in the synthesis (Figure 7). The tops and edges of the polygonal nanoplates had to be initially rounded and shortened, causing the solution to briefly turn violet again. Eventually, the color of the solution returned to blue, shifting the hue slightly towards green (turquoise), and the edges and tops of the polygonal nanoplates were rebuilt. As many samples were taken from also many separately synthesized turquoise MA2 colloids, different steps of this transition after NaOH addition are captured in the TEM images. For example, in Figure 3 b3, smaller nanoparticles sticking to larger ones still resemble 10 nm polyhedra observed in the MA1 colloid; in Figure 3 b2, their dimensions are smaller, while in Figure 3 b4 the only visible addition to large NPs is a thin layer of silver. All of these Ag residues could be those dissolved to some degree in polyhedra originating from the Ostwald ripening process described above. It is also probable that these Ag residues are created by dissolving the tops of triangle nanoplates after NaOH addition, which could not be fully rebuilt at subsequent stages of synthesis. Since TEM images could only be obtained for colloids in the stable final stage, i.e., after NaOH addition, it is not possible to determine whether Ag residues were already present in the MA2 colloid before NaOH addition. Both explanations for appearance of those Ag residues must therefore be taken into account (question mark in Figure 7).

In contrast to MA2, in the syntheses of MA3 and MA4, decreasing the degree of ME protonation by NaOH addition enabled the complete deconstruction of the MA3 and MA4 colloids into one dominated by polyhedra. The pH increase also moved the process back one step (from a violet to orange solution) to finally complete the entire synthesis, passing through the violet step again and ending with the final green step. Importantly, in the synthesis of MA3 and MA4 there was no blue stage, corresponding to the dominance of nanoplates, even after NaOH addition. The polyhedral shape of the NPs was determined by the thermodynamic mechanism of NP growth. Ultimately, in the MA4 synthesis, where the amount of added seeds was larger, the nanoparticles aggregated into beads. Combining the above observations and conclusions, the function of mellitic acid as a capping agent cannot be attributed to the adsorption of single mellitic acid molecules on the surface of Ag {111} but rather to the adsorption of acid molecules cross-linked by hydrogen bonds on the surface of Ag {111}. The network created in this way provides template-like support for thermodynamically unstable nanoplates, inhibits the formation of polyhedral NPs, and helps destabilize those created previously. The increased deprotonation of MA molecules did not prevent the formation of the hydrogen bond network but instead made it less dense. Through the resulting “holes”, the evolution of NPs from nanoplates into polyhedra becomes possible, but only at a sufficiently low MA/Ag^+^ ratio in MA3 and MA4 syntheses.

### SERS Substrate Test

In order to test the effectiveness of the obtained colloids (MA1, MA2, and MA4) as SERS substrates, Raman spectra of a mixture of the colloid and a solution of 4-mercaptobenzoic acid (4-MBA) with a final 1 mM concentration were recorded. 4-MBA as a compound was a thoughtful choice for this enhancement test. Many studies in the literature demonstrating the SERS effect on different substrates used compounds with a very high scattering cross-section, usually dyes, e.g., rhodamine, whose small amounts in solutions are easily detected due to the resonance effect. The compound used in this work is a simple aromatic thiol whose Raman spectra are not resonantly enhanced.

To assess the enhancement, the SERS spectra of 1 mM 4-MBA in our colloids were compared with the SERS spectrum of 1 mM 4-MBA in the colloid of spherical Ag nanoparticles synthesized with hydroxylamine hydrochloride as a reducer.^94^ This reference colloid was chosen as one that is widely used due to its high enhancement properties and fast and easy synthesis. The test used in this work enabled us to determine in a direct way if a better SERS substrate had been synthesized than the one already known and used.

**Figure 8:**
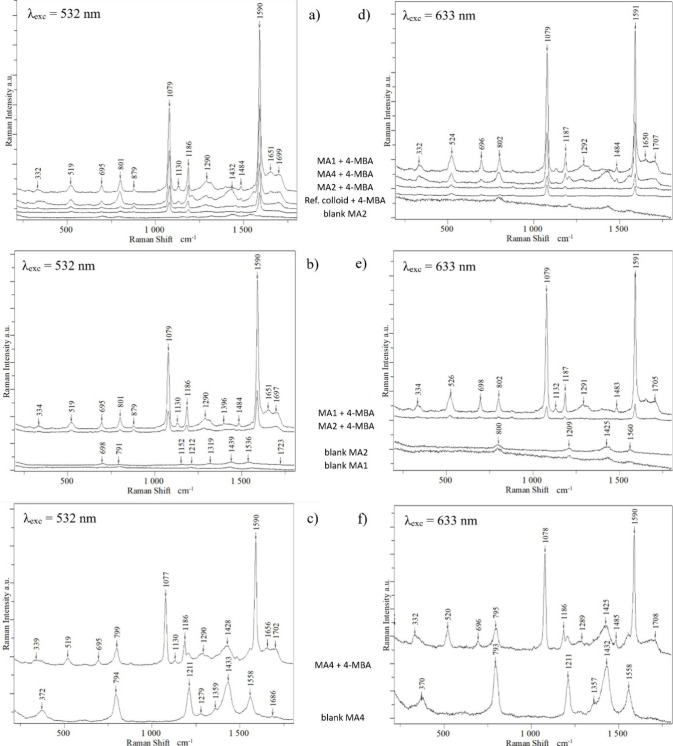
SERS spectra of blank MA-capped colloids and 1 mM 4-MBA recorded in MA-capped and hydroxylamine-reduced colloids using (a–c) λ = 532 nm and (d–f) λ = 633 nm. Spectra are arranged sequentially from top to bottom on the picture in the following order: (a and d) MA1, MA4, MA2, hydroxylamine-reduced, blank MA2; (b and e) MA1, MA2, blank MA1, blank MA2; and (c and f) MA4, blank MA4.

As can be seen in Figure 8a and d, all tested MA-capped colloids enhance the Raman spectrum of 4-MBA more strongly than the reference hydroxylamine-reduced AgNP colloid. Table 1 compares the intensity ratios of the 1079 cm^–1^ band in the SERS spectrum of 4-MBA recorded using MA-capped colloids to the intensity of this band recorded on the reference colloid.

**Table 1:** Intensity Ratio of 1079 cm^–1^ Band in the 4-MBA SERS Spectra^a^

λ_exc_ (nm)	MA1/hydroxylamine-reduced	MA2/hydroxylamine-reduced	MA4/hydroxylamine-reduced
532	8	2.2	5.1
633	11.3	1.2	4.7

a Recorded in MA-capped (MA1, MA2, and MA4) and hydroxylamine-reduced colloids with two laser lines (532 and 633 nm).

The greatest relative increase in enhancement (8–11×) was observed for MA1 colloids. Next is the MA4 dark green colloid. Nevertheless, this colloid revealed a significant disadvantage as a SERS substrate: it has its own relatively strong SERS spectrum (“blank MA” in Figure 8c and f). It is probably a spectrum of mellitic acid (MA) and/or ascorbic acid (AA) in a specific configuration, captured when their molecules are trapped between aggregated NPs (Figure 3 d). However, it should be stressed that we were not able to record reference SERS spectra of MA and AA, separately or in the mixture, which would resemble the spectrum recorded in the pure MA4 colloid. In the blank MA1 and MA2 colloids, this spectrum was also observed, but its intensity was negligibly low (Figure 8 a–e). This feature and the high SERS enhancement prove the applicability of MA1 and MA2 colloids as efficient SERS substrates.

**Figure 9:**
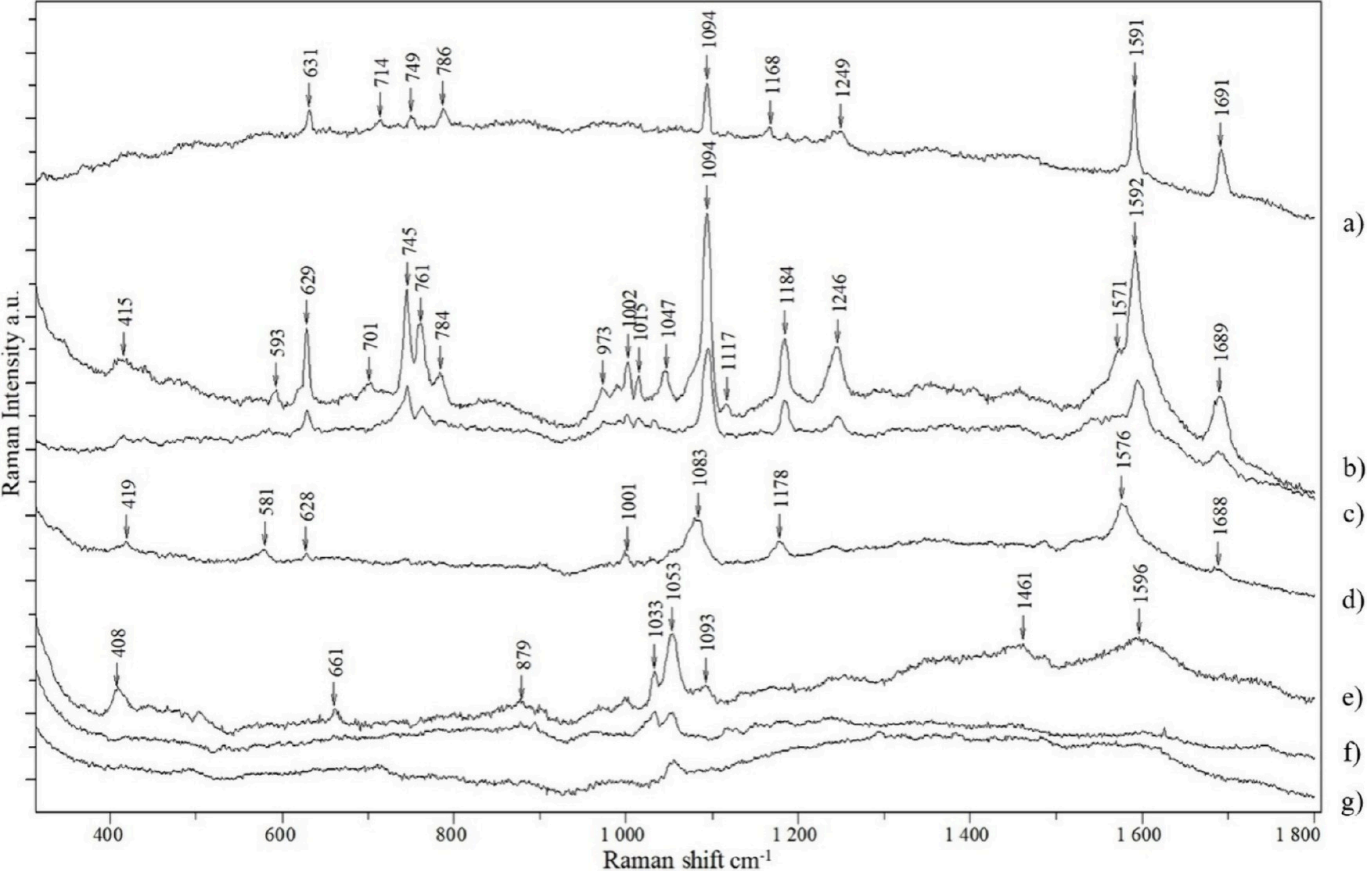
Spectra of 4-chloromethcathinone (4-CMC). (a) Normal Raman spectrum of powder. SERS spectra of the MA1 colloid with 4-CMC with concentrations (b) 10^–5^ , (c) 10^–6^, (d) 10^–7^, (e and f) 10^–8^, and (g) 10^–9^ M.

In order to demonstrate the practical applications of the synthesized colloids, a 4-chloromethcathinone (4-CMC, clephedrone) detection limit test was performed. This compound belongs to the class of psychoactive stimulants, so-called designer drugs. To increase the detection of trace amounts of this illegal and dangerous substance, it is necessary to use a very sensitive method that will also allow for clear identification. The MA1 colloid was selected for testing as it gave the best results in the 4-MBA tests described above. The experimental results are summarized in Figure 9. The topmost spectrum (a) is the Raman spectrum of 4-CMC powder, and the next spectra below are SERS spectra of 4-CMC with final concentrations from 10^–5^ to 10^–9^ M. The SERS spectra of 4-CMC with *C* = 10^–5^ and 10^–6^ M (Figure 9b and c) contain numerous bands distinguishing this drug from its derivatives. In the spectrum of the sample with *C* = 10^–7^ M (Figure 9 d), the characteristic bands are still detectable, but a slight shift can be observed in relation to the previous spectra. The spectra of subsequent samples with *C* = 10^–8^ M (Figure 9e and f) differ significantly from those of more concentrated samples. Additionally, repeatedly recording spectra from different places in the sample showed their heterogeneity, changes in the intensity ratio of the 1033 and 1053 cm^–1^ bands, and the disappearance of the 408 cm^–1^ band. Shifts of the bands, the disappearance of some, and the formation of others may indicate a change in the structure of 4-CMC molecules on the Ag surface as a result of reduced surface coverage. Knowledge of the SERS spectra at the lowest concentrations is therefore crucial use in detecting trace amounts of this compound; the Raman spectrum of the powder is no longer useful here. The detection limit of the spectrum of this compound was reached at a concentration of 10^–9^ M (Figure 9 g), although it is difficult to call this spectrum diagnostic. The SERS spectra presented above are, to the best of our knowledge, the first SERS studies of this designer drug.

## Conclusions

The analysis of TEM images and UV–vis excitation spectra of four obtained colloids showed that all syntheses using mellitic acid had a common beginning consisting of the formation of polyhedra (decahedra and icosahedra) and nanoplates (disks, triangles, and hexagons). Further synthesis paths were diversified at subsequent stages. At a sufficiently high concentration ratio of MA/Ag^+^ and at the same time a sufficiently high Ag^+^ concentration (MA2 synthesis), the synthesis path was dominated by the kinetic mechanism. This enabled the growth and sharpening of the tops of triangular and hexagonal nanoplates and a significant increase in their percentage contribution. The Ostwald ripening process, probably responsible for the latter, left remnants of unetched polyhedra in the MA2 colloid. A slight increase in pH at the final stages of the synthesis of MA3 and MA4 ultimately promoted the formation of colloids composed mostly of polyhedra. In the case of the colloid with a larger number of seeds (MA4), partial aggregation of NPs occurred.

The analysis of the synthesis process enabled us to deduce the mechanism of the interaction of mellitic acid as a capping agent. MA molecules most likely form a network of hydrogen bonds that preferentially coat the {111} facets of the resulting NPs. Due to its two-dimensional structure, this network promotes the formation of two-dimensional NP structures as well. At a sufficient Ag^+^ concentration, the effectiveness of protection against the growth of polyhedral planes decreases with subsequent MA deprotonation.

The SERS enhancement test has shown a considerable increase in the intensity of the Raman signals on synthesized NPs as compared with the hydroxylamine-reduced colloid most frequently used in SERS measurements. The second SERS test showed that the MA1 colloid enables the detection and identification of trace amounts of 4-chloromethcathinone (4-CMC, clephedrone), with a detection limit of 10^–9^ M. Additionally, for the first time, SERS spectra of this designer drug and their dependence on concentration are presented.

As a result, the development of a green, fast, and inexpensive method for producing new SERS substrates was finally demonstrated.
